# Introgressed Animal Schistosomes *Schistosoma curassoni* and *S. bovis* Naturally Infecting Humans

**DOI:** 10.3201/eid2212.160644

**Published:** 2016-12

**Authors:** Elsa Léger, Amadou Garba, Amina A. Hamidou, Bonnie L. Webster, Tom Pennance, David Rollinson, Joanne P. Webster

**Affiliations:** Royal Veterinary College, University of London, London, UK (E. Léger, T. Pennance, J.P. Webster);; RISEAL Niger, Niamey, Niger (A. Garba, A.A. Hamidou);; Natural History Museum, London (B.L. Webster, D. Rollinson)

**Keywords:** schistosomes, schistosomiasis, introgression, Niger, hybrids, zoonotic hybrid species, parasites, parasitic worms, Schistosoma curassoni, Schistosoma bovis, Schistosoma haematobium, zoonoses, humans, cattle, livestock, neglected tropical disease, Africa

**To the Editor:** Schistosomiasis, a disease caused by infection with parasitic worms (schistosomes), is a neglected tropical disease across many parts of the world. Numbers of infected livestock are unknown, but >250 million persons are infected; the greatest number of cases are in sub-Saharan Africa (*1*). Schistosome eggs are excreted through urine or feces, depending on the species, and hatch into miracidia upon contact with freshwater. Larvae are transmitted to the mammalian host indirectly through a molluscan intermediate host. Goals to eliminate schistosomiasis by 2020 in select countries in Africa have been proposed by the World Health Organization (http://www.who.int/neglected_diseases/NTD_RoadMap_2012_Fullversion.pdf) and a coalition of partners combating neglected tropical diseases (*2*).

Selective pressures imposed by natural phenomena and human activities affect the dynamics and distribution of schistosomiasis. For example, ongoing changes in agricultural practices in some regions are creating new water bodies shared by humans and domestic livestock. These anthropogenic changes increase opportunities for mixing of and subsequent exposure to different *Schistosoma* species, especially those that infect humans and livestock. Such mixing could increase the potential for novel zoonotic hybrid parasites to emerge and become established (*3**,**4*).

Since the 1940s, researchers have suspected that natural hybridization within and between human and animal schistosome species occurs in definitive and intermediate hosts. In western Africa, hybrids, predominantly between *S. haematobium* (human schistosome) and *S. bovis* or *S. curassoni* (livestock schistosomes), have been isolated from children and snails (*5*,*6*). Hybrids between these livestock-only *Schistosoma* species have also been reported in cattle and sheep but not in other hosts (*7*). As demonstrated in the field and under experimental laboratory conditions, neither *S. bovis* nor *S. curassoni,* as single species, can fully develop in humans or nonhuman primates (*8*,*9*).

We report evidence of a child in Niger who was infected by livestock-specific schistosomes through the hybridization and introgression of *S. bovis* with *S. curassoni*. Samples were collected from a 10-year-old girl in Kokourou, Tillaberi region, Niger (14°20′61.50′′N, 0°91′94.0′′E), as part of longitudinal monitoring and evaluation of national disease control interventions in the area. Tillaberi has an ongoing high prevalence and infection intensity of schistosomiasis, despite more than a decade of high-coverage mass treatment with praziquantel.

Schistosoma eggs were recovered from the child’s urine. Miracidia that hatched from the eggs were stored on a Whatman FTA card (Sigma-Aldrich, St. Louis, MO, USA), providing the opportunity to perform noninvasive molecular characterization on schistosome larvae without any necessity for laboratory passage. Multilocus analyses of mitochondrial and nuclear DNA regions (*6*) on all 42 larvae collected from the child identified 2 individual miracidia with a livestock *S. bovis* × *S. curassoni* hybrid profile with no genetic signal from human-specific *S. haematobium*. Although no species-specific markers exist for determining parental lineages, internal transcribed spacer (ITS), a powerful marker for detecting introgression, has been used successfully to detect hybridization events across the *Schistosoma* genus. A partial mitochondrial *cox*1 sequence for miracidia was identical to that for *S. bovis*, and the nuclear ITS and partial 18S rDNA sequences were identified as *S. curassoni*. 

One miracidium from the child had a pure *S. haematobium* profile, 13 had *S. bovis* (*cox*1) × *S. haematobium* (ITS) profiles, and 5 had *S. bovis* (*cox*1) × *S. haematobium* × *S. curassoni* (ITS) profiles, suggesting the potential for repeated interactions and cross-pairings among these 3 species. No *S. curassoni* mitochondrial DNA was found in miracidia. The molecular data suggest that these hybrids were not first generation, but a result of parental and/or hybrid backcrosses. The data confirm the occurrence of bidirectional introgression between *Schistosoma* species that infect livestock and those that infect humans in Niger. Our data also show that hybrid livestock:livestock schistosomes can directly infect humans without combining with a *Schistosoma* species that infects humans. 

Hybridization of parasites is an emerging public health concern at the interface of infectious disease biology and evolution (*3*). Our results raise several critical questions regarding the evolution, epidemiology, health effects, and ultimate control of schistosomiasis. Hybrid schistosomes, and, in particular, hybridized livestock:livestock schistosomes infecting humans, could potentially extend intermediate and definitive host ranges; confer altered infectivity, virulence, and drug efficacy; or even potentially replace existing single species (*3**,**4*). Hybrid vigor has been observed experimentally for schistosomes, and similar evidence is gathering from experiments with other parasites. Our results strongly indicate that hybrid livestock:livestock schistosomes can infect a human definitive host, even when neither of its single parental livestock species appears compatible. Such novel livestock schistosome hybrids infecting humans could be predicted to spread to wherever suitable intermediate snail hosts are endemic, as has recently been reported for zoonotic *Schistosoma* hybrids in Corsica (*10*).

It is imperative to identify and understand the transmission dynamics of introgressed *Schistosoma* species combinations. The detection of multiple introgressed hybrids with mixed ancestry in a single child suggests that *Schistosoma* species may be adapting to recent anthropogenic changes. If novel zoonotic hybrid species are playing a role in maintaining and exacerbating schistosome transmission, illness caused by infection, or both, treatments for humans and livestock may have to be adjusted accordingly within a One Health framework (*4*).
